# Supplementary material for: Identification of photoperiod-induced specific miRNAs in the adrenal glands of Sunite sheep (Ovis aries)
Source: Front Vet Sci. 2022 Jul 22;9:888207. doi: 10.3389/fvets.2022.888207 (PMC9354845; doi:10.3389/fvets.2022.888207)
Supplement: Supplementary file 1 [file Data_Sheet_1.ZIP › Supplementary file/miRDeep2 file.zip!/miRDeep2 file/Know_Structure/SPLP42A3/oar-mir-154b.pdf]

miRBase precursor : oar-mir-154b
Total read count : 1013
oar-miR-154b-5p read count : 969
oar-miR-154b-3p read count : 44
remaining reads : 0

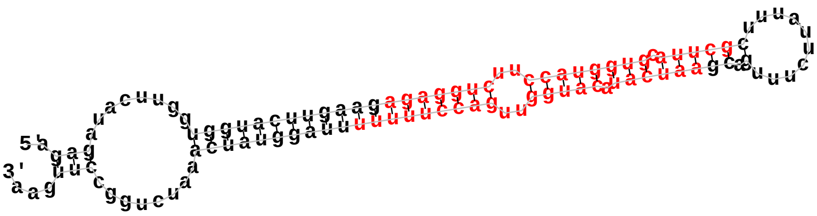

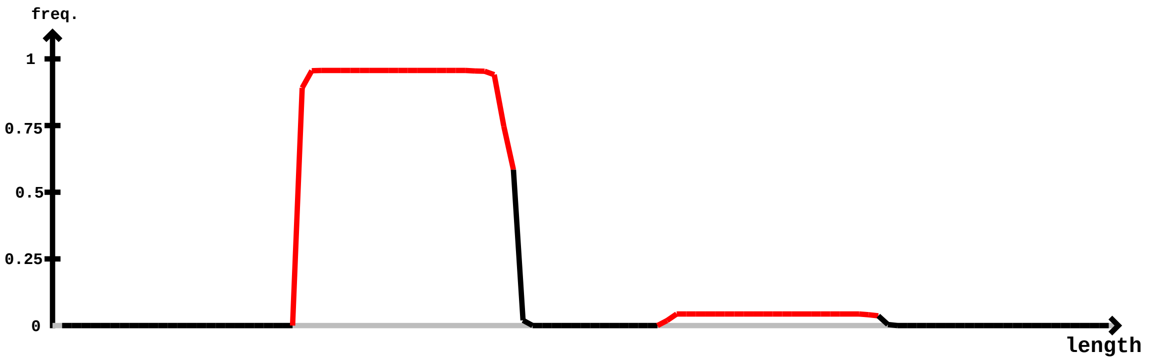

oar-miR-154b-3p
oar-miR-154b-5p

```
5'- agagauacuuggugguacuugaagagaggucuuccauggugcauucgcuuuauucuuugacgaaucauacagguugaccuuuuuuuagguaucaaaucuggccuugaa   -3'    exp
    .(((........((((((((((((((((((..((((((((.((((((..........).))))))))).))))).))))))))))))))))))........)))...    reads      mm          sample
    ........................agaggucuuccauggugc..................................................................    2          0           seq
    ........................agaggucuuccauggugca.................................................................    1          0           seq
    ........................agaggucuuccauggugcau................................................................    11         0           seq
    ........................agaggucuuccauggugcauu...............................................................    188        0           seq
    ........................agaggucuuccauggugcauuc..............................................................    162        0           seq
    ........................agaggucuuccauggugcauucg.............................................................    519        0           seq
    ........................agaggucuuccauggugcauucgc............................................................    20         0           seq
    .........................gaggucuuccauggugcau................................................................    2          0           seq
    .........................gaggucuuccauggugcauu...............................................................    8          0           seq
    .........................gaggucuuccauggugcauuc..............................................................    2          0           seq
    .........................gaggucuuccauggugcauucg.............................................................    53         0           seq
    ..........................aggucuuccauggugcauuc..............................................................    1          0           seq
    ..............................................................aaucauacagguugaccuuu..........................    3          0           seq
    ..............................................................aaucauacagguugaccuuuu.........................    2          0           seq
    ..............................................................aaucauacagguugaccuuuuu........................    14         0           seq
    ...............................................................aucauacagguugaccuuu..........................    2          0           seq
    ...............................................................aucauacagguugaccuuuuu........................    19         0           seq
    ...............................................................aucauacagguugaccuuuuuu.......................    4          0           seq
```
